# Effect of a Community Gerontology Program on the Control of Metabolic Syndrome in Mexican Older Adults

**DOI:** 10.3390/healthcare10030466

**Published:** 2022-03-02

**Authors:** Víctor Manuel Mendoza-Núñez, Gabriela Pulido-Castillo, Elsa Correa-Muñoz, Juana Rosado-Pérez

**Affiliations:** 1 Research Unit on Gerontology, Faculty of Higher Studies Zaragoza, National Autonomous University of Mexico (UNAM), Mexico City 09230, Mexico; elcomm_unam@yahoo.com.mx (E.C.-M.); juanarosadoperez@gmail.com (J.R.-P.); 2 Master of Nursing, Faculty of Higher Studies Zaragoza, National Autonomous University of Mexico (UNAM), Mexico City 09230, Mexico; menuvi05@yahoo.com.mx

**Keywords:** metabolic syndrome, older adults, community gerontology model, healthy aging

## Abstract

Background: Metabolic syndrome (MS) is highly prevalent in older adults; it constitutes a risk factor for cognitive deterioration, frailty, and Alzheimer’s disease. For this reason, the WHO has pointed out the importance of the implementation of community programs for the training of healthy aging. The aim of this study was to evaluate the effect of a community gerontology program framed in active aging on the control of metabolic syndrome in older adults. Methods: An experimental study was carried out in a convenience sample of 80 older adults diagnosed with MS according to the ATPIII criteria, comprising (1) experimental group (EG), *n* = 40; (2) control group (CG), *n* = 40. During a 6-month period, the EG participated in a supervised community gerontology program, and the CG was assessed monthly. Results: A statistically significant decrease was observed in the number of components for the diagnosis of MS. In this regard, of the total of participants with a diagnosis of MS in EG, only 28% maintained the diagnosis of MS (ATPIII ≥ 3 criteria), in contrast to 83% of the CG participants (*p* < 0.0001). Conclusions: Our findings suggest that health self-care training within the framework of active aging is effective for the control of MS in older adults.

## 1. Introduction

The metabolic syndrome (MS) is a group of biochemical and clinical alterations characterized by insulin resistance, dyslipidemia, inflammation, coagulation disorders, obesity, and arterial hypertension, which is why it constitutes a risk factor for cardiovascular diseases (CVD), type 2 diabetes mellitus (DM2) and all causes of death [1]. There are different criteria for the diagnosis of MS, among the most used are those established by (1) National Cholesterol Education Program Adult Treatment Panel III (ATP III), (2) International Diabetes Federation (IDF), (3) American Association of Clinical Endocrinologists (AACE), (4) World Health Organization (WHO), and (5) American Heart Association/National Heart, Lung, and Blood Institute (AHA/NHLBI) [1,2,3]. In this context, in 2009, a harmonized definition was proposed, establishing that MS could be diagnosed when three of the following five risk factors are present: waist circumference augmented with specific criteria of the population and the country; triglycerides ≥ 150 mg/dL, HDL-C < 40 mg/dL in men and <50 mg/dL in women, systolic blood pressure ≥ 130 mm Hg or diastolic blood pressure ≥ 85 mm Hg and fasting glucose > 100 mg/dL, with the inclusion of patients taking medication to manage hypertriglyceridemia, low HDL-C, hypertension, and hyperglycemia [4].

The prevalence of MS depends on the criteria adopted in each study. In this regard, a meta-analysis carried out by Gutiérrez-Solís et al. (2018) in the Mexican adult population reported a prevalence of MS of 41%, although the frequency ranged between 31% and 54%, according to the definition of MS used. The prevalence was 54% with the IDF criteria, 48% with the AHA/NHLBI criteria, 31% with the ATP III criteria, and 31% with the WHO criteria. The results of this study revealed that the prevalence of MS in Mexico is higher than that reported in the United States (34.2%) and in Latin America (24.9%) [5].

On the other hand, it has been found that aging per se constitutes a risk factor for developing MS, due to the morphological, physiological, and biochemical changes that occur during the process. MS, in turn, is considered a model of accelerated or premature aging [6]. Nevertheless, it is important to note that sociocultural aspects are also decisive since the reported prevalence of MS 23.8% in older adults in Turkey (Cankurtaran et al., 2006) contrasts with 50% found by Aguilar-Salinas et al. (2004) for those over 60 years of age with the ATPIII criteria [7,8]. In comparison, a prevalence of MS of 72.9% (75.7% men and 70.4% women) has been reported in Mexican older adults (≥65 years), applying AHA/NHLBI/IDF criteria. The most frequent alterations were found according to the MS criteria, and cutoff points were arterial hypertension (92.4%), hypertriglyceridemia (77.8%), low HDL-cholesterol, or hyperglycemia (77.1%), and central obesity (65.4%) [9].

Within the framework of preventive medicine, it is important to highlight the main outcomes or consequences of MS linked to old age. In this regard, an increase in the risk of several alterations has been reported, including decreased muscle strength and sarcopenia, frailty, cognitive deficits, sleep disturbances, multiple chemical sensitivity, sensorineural hearing loss, kidney stone disease, etc. [10,11,12,13,14,15,16,17,18].

In this context, several studies have shown that the practice of healthy lifestyles (physical exercise and diet) is effective in controlling MS [19,20]. Therefore, our research group has developed a model of gerontological nuclei (mutual help groups of older adults with training in self-care) for the implementation of community programs for healthy aging, demonstrating their effectiveness in glycemic control, cognitive function, and oxidative stress [21,22,23]. For this reason, the objective of the present study was to evaluate the community gerontology program of gerontological nuclei on the control of MS in Mexican older adults.

## 2. Materials and Methods

**Figure 1:**
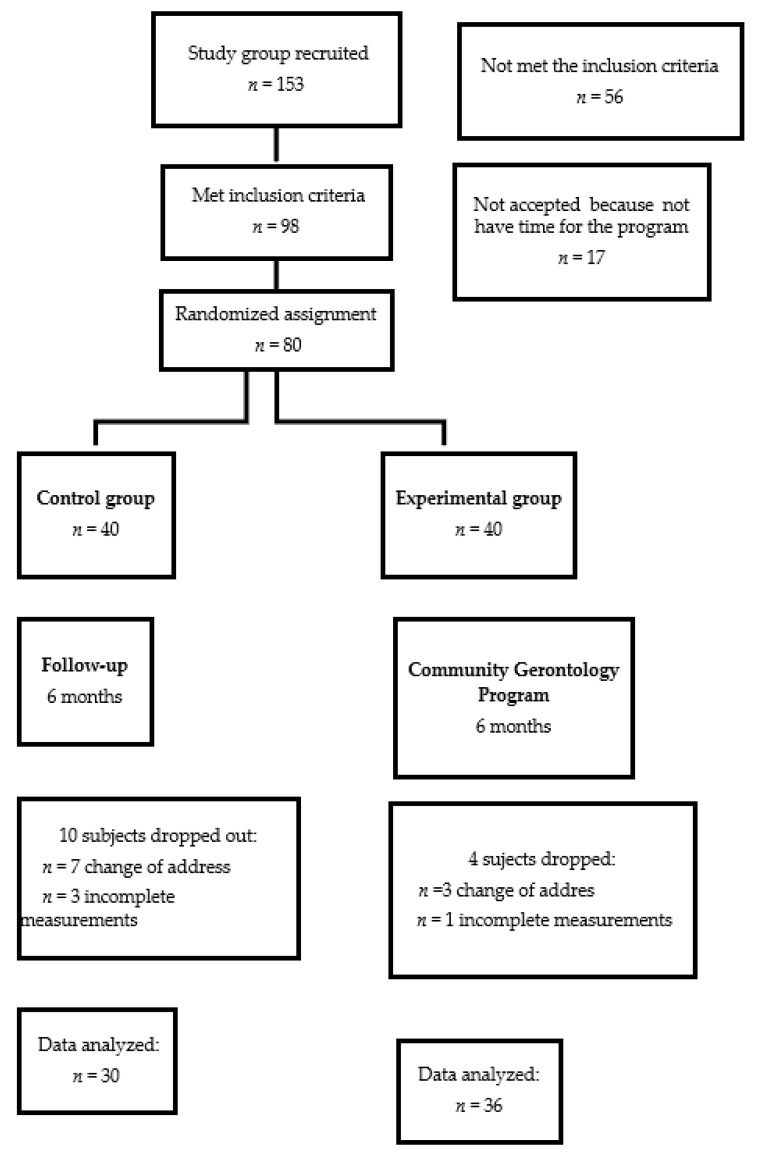
General diagram of the study.

With prior informed consent, an experimental study was carried out in a convenience sample of 80 adults aged 60 to 69 years with a diagnosis of MS without comorbidity (Figure 1). A random assignment was made for the conformation of the two groups of (1) experimental (EG, *n* = 40) and (2) control (CG, *n* = 40), during a 6-month period. The EG participated in a supervised community gerontology program (CGP), and the CG participants attended weekly meetings of recreational activities (board games or painting workshop or storytelling) of two hours. The groups were reunited at different schedules to avoid communication between them. All participants were assessed monthly, with anthropometric measurements, blood pressure, and received guidance on healthy lifestyles (diet and physical exercise).

Elimination criteria were established as 20% absence from the EG program sessions and absence from two or more CG monthly follow-up appointments. The Ethics Committee of the “Universidad Nacional Autónoma de México (UNAM) Zaragoza Campus” approved the research protocol for this study (PAPIIT IN218718).

The following measurements were performed on all participants before and after 6 months from the start of the study.

### 2.1. Clinical Assessment

A comprehensive clinical evaluation was carried out to rule out other chronic non-communicable diseases, cognitive impairment, and depression, for which, in addition to the clinical history, the Folstein Mini-Mental Examination and the Yesavage Geriatric Depression Scale were used as screening instruments. Anthropometric measurements and blood pressure (BP), systolic blood pressure (SBP), and diastolic blood pressure (DBP) were also performed according to a standardized protocol [24].

### 2.2. Metabolic Syndrome Diagnosis

Metabolic syndrome was diagnosed according to the del (NCEP/ATP III) criteria, if the people met three or more of the following five variables: (1) waist circumference (≥102 cm for men or ≥88 cm for women); (2) serum triglycerides ≥ 150 mg/dL; (3) HDL cholesterol < 40 mg/dL in men and <50 mg/dL in women; (4) BP ≥ 130/85 mm Hg; (5) fasting serum glucose level ≥ 100 mg/dL. Treatment with antidiabetic, antihypertensive, or triglyceride-lowering drugs was considered a positive variable [25].

### 2.3. Biochemical Measurements

Blood samples were collected from the participants by venipuncture in vacuum tubes (Beckton-Dickinson, Mexico City, Mexico), with a previous 8 h fast to determine glucose, HDL cholesterol and triglycerides, urea, uric acid, albumin, and C-reactive protein. In all cases, commercial kits (Randox Laboratories Ltd., Crumlin, London, UK) were used.

### 2.4. Community Gerontology Program (CGP)

**Figure 2:**
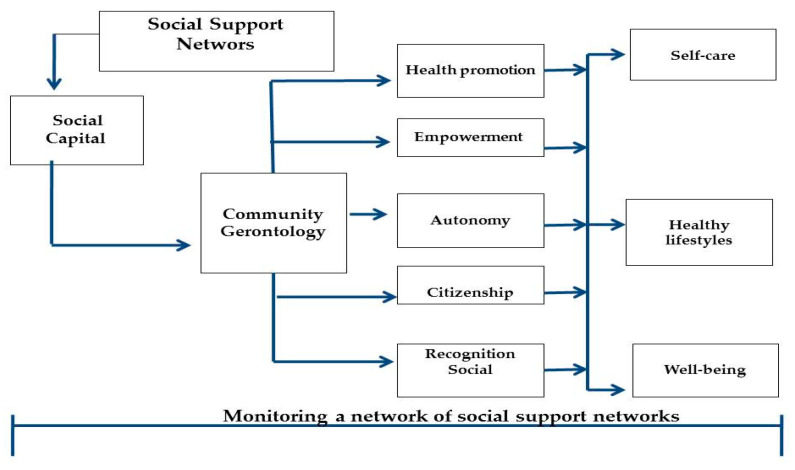
Characteristics of the community gerontology program (CGP). The key element of the CGP is the formation and optimal use of social support networks with the purpose of integrating a gerontological social capital, to develop community gerontology programs, establishing as pillars the promotion of health, empowerment, autonomy, citizenship, and social recognition to adopt self-care for healthy aging through the adoption and strengthening of healthy lifestyles, to maintain, prolong, or recover physical, psychological, and social functioning, within the framework of the organization and monitoring of a “network of social support networks for healthy aging”.

The CGP was carried out during the months of April and July 2019, considering the pre- and post-intervention measurements. This was based on the paradigm of active aging, whose purpose is to develop and strengthen the empowerment of older adults to acquire knowledge and skills for healthy aging. In this regard, the Faculty of Higher Studies Zaragoza, of the National Autonomous University of Mexico, has developed a model of gerontological nuclei (self-care and peer-help), through the training of older adults for self-care, mutual aid, and self-management (Figure 2) [26]. For this purpose, a workshop course was organized with a constructivist approach so that older adults could acquire practical and significant knowledge for healthy aging. The CGP is made up of 20 weekly theoretical-practical sessions of 2 h (40 h in total) (Table 1), in which a popularization book “Active and Healthy Aging” was used as support material, prepared expressly for people older adults, with accessible language, large print, with diagrams and demonstrative vignettes, ruled by experts in community gerontology [27].

### 2.5. Statistical Analysis

Percentages, as well as mean and standard deviation, were calculated; data were analyzed using ANOVA for repeated measures, chi-squared test to compare proportions, and McNemar’s test to compare the effect of proportions of related samples. For this purpose, the statistical program SPSS V 20 was used.

## 3. Results

**Figure 3:**
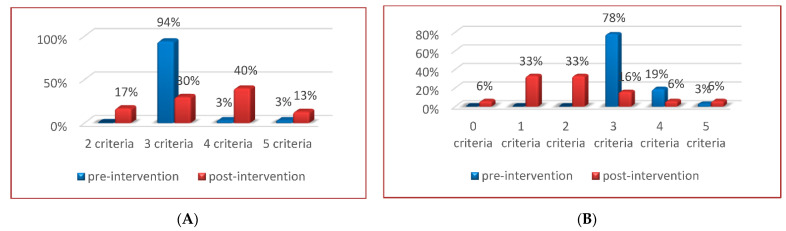
Percentage of the number of MS criteria pre and post-intervention. In (**A**) (control group) pre-intervention, 28/30 (94%) subjects had 3 MS criteria, 1/30 (3%) had 4 MS criteria, and 1/30 (3%) had 5 MS criteria; post-intervention, 5/30 subjects (17%) changed from 3 to 2 MS criteria, 28/30 (94%) changed to 9/30 (30%) with 3 MS criteria, 1/30 (3%) changed to 12/30 (40%) with 4 MS criteria and 1/30 (3%) changed to 4/30 (13%) with 5 MS criteria. In (**B**) (experimental group), pre-intervention 28/36 (78%) subjects had 3 MS criteria, 7/36 (19%) had 4 MS criteria, and 1/36 (3%) had 5 MS criteria; post-intervention, 2/36 subjects (6%) changed from 3 to 0 MS criteria, 12/36 (33%) changed from 3 to 1 MS criteria, 12/36 (33%) changed from 3 to 2 MS criteria, 28/36 (78%) changed to 6/36 (16%) 3 MS criteria, 7/36 (19%) changed to 2/36 (6%) with 4 MS criteria, and 1/36 (3%) changed to 2/36 (6%) with 5 MS criteria. Therefore, 25/30 (83%) of the control group, in comparison with 10/36 (28%) of the experimental group, maintained a diagnosis of MS (3 or more components) after the intervention, whose difference was statistically significant (*p* < 0.001); (**A**) control group; (**B**) experimental group.

Table 2 shows the sociodemographic characteristics of the population by study group. Regarding the effect of the CGP, a statistically significant positive change was observed in the number of components for the diagnosis of MS. Of the total of participants with a diagnosis of MS in EG, only 28% maintained the criteria (≥3) for said diagnosis, in contrast to 83% of participants in CG (*p* < 0.0001). Likewise, 40% of the CG participants continued with four criteria of MS, compared with 16% of the EG participants (*p* < 0.001) (Figure 3). A statistically significant decrease was also observed in the average number of MS criteria in EG, compared with CG (EG: baseline, 3.2 ± 0.5 post-intervention 2 ± 1.2; CG: baseline, 3.1 ± 0.4 vs. post-intervention, 3.5 ± 0.9, *p* < 0.001) (Table 3).

After the community intervention, a statistically significant decrease in BMI was observed in the EG group, compared with the CG group (EG: baseline, 32 ± 6 vs. post-intervention, 30 ± 5, *p* < 0.01; CG: baseline, 28 ± 5 vs. post-intervention, 29 ± 3). Similarly, SBP (SG: baseline, 129 ± 14 vs. post-intervention, 126 ± 20, *p* < 0.05; CG: baseline, 128 ± 16 vs. post-intervention, 132 ± 10, *p* < 0.05) and DBP (CG: baseline, 82 ± 8 vs. post-intervention, 85 ± 9; EG: baseline, 85 ± 11 vs. post-intervention, 81 ± 13, *p* < 0.05) showed a statistically significant decrease in EG, compared with CG (Table 4).

Regarding the biochemical parameters, a statistically significant increase in the concentration of HDL cholesterol in EG participants was found, compared with that in CG participants (EG: baseline, 56 ± 16 vs. post-intervention, 63 ± 17; CG: baseline, 52 ± 12 vs. post-intervention, 46 ± 9, *p* < 0.001), coupled with a decrease in blood triglyceride concentration (EG: baseline, 177 ± 61 vs. post-intervention, 139 ± 43; CG: baseline, 192 ± 78 vs. post-intervention, 200 ± 90, *p* < 0.01) (Table 5).

In relation to the number of subjects with values above the cutoff point for each MS component, a statistically significant decrease was observed in the percentage of people with high blood pressure in EG, in contrast to CG (EG: baseline, 61% vs. post-intervention, 28%; CG: baseline, 47% vs. post-intervention, 60%, *p* < 0.01), as well as with high triglycerides (EG: baseline, 75% vs. post-intervention, 64%; CG: baseline, 87% vs. post-intervention, 80%; *p* < 0.01) and low HDL concentration (EG: baseline, 44% vs. post-intervention, 17%; CG: baseline, 43% vs. post-intervention, 70%, *p* < 0.05) (Table 6).

## 4. Discussion

The detection, prevention, and treatment of MS are of great relevance in old age, due to its magnitude and the high risk for diabetes mellitus, cardiovascular diseases, neoplastic diseases, cognitive deterioration, and Alzheimer’s disease (AD). In relation to this, the concept of “diabetes mellitus type 3” or “diabetes of the brain” has been proposed, to highlight the relationship between the pathophysiological process of DM2 and AD linked to MS, considering that hyperglycemia, dyslipidemia, insulin resistance, oxidative stress, and chronic inflammation caused by MS influence and enhanced by aging (inflamm-aging) occurs in both diseases [28,29,30].

A community strategy for healthy aging is the “peer social support” (PSS), linked to social support networks as a key element of gerontological social capital. In this sense, the PSS implies support between people who share similar experiences, due to their age, health status, and characteristics that allow them to establish a relationship between equals [31], for example, the gerontological nuclei, as proposed in our community program of gerontology. In this regard, positive results have been reported in community social support programs among peers for the control of diabetes mellitus in adults, where four main elements of peer support required by adults with diabetes will be found: (1) exchange of experiences, (2) cohesion and collectivity, (3) trust and reciprocity, and (4) social participation [32,33].

In this context, as a field of study oriented to the planning, organization, and development of strategies for the implementation of intervention programs that allow achieving the maximum health, well-being, and quality of life of aging and aging people in their environment through personal and “peer-to-peer” self-care, community gerontology recognizes the potential of functional older adults as human and social capital for their own development [34].

The WHO has highlighted the importance of the implementation of community programs for healthy aging in order to develop the empowerment of older adults to adopt, adapt, and strengthen healthy lifestyles to prevent and control chronic non-communicable diseases, as well as maintain physical, mental, and social functionality as long as possible [35,36].

Among the strategies to achieve this objective, the development of gerontological care models at the community level framed in active aging has been proposed, considering the training of older adults for their personal and “peer-to-peer” self-care as a key element. For this reason, in the present study, the effect of a community healthy aging program developed by our working group was evaluated.

Among the most relevant results of this study, it was found that 7 out of 10 older adults diagnosed with MS in EG had an improvement in some clinical and biochemical parameters after the community intervention program. Therefore, of the 100% of older adults diagnosed with MS before participating in the program, only 28% maintained this diagnosis post-intervention, in contrast to 83% of the CG participants. Likewise, before the community intervention, 19% of the EG participants had four criteria for MS, and with the training of the healthy aging program, this changed to 6%; in contrast, at the beginning of the measurement, 3% of the CG participants had four criteria, and at 6-month follow-up, this percentage changed to 40%. The overall quantitative estimate of effect was 37%.

These findings may reflect the effect of community programs on therapeutic adherence in supervised community groups, since the CG participants, although receiving health education and a periodic clinical and anthropometric evaluation (monthly), did not show the effect of verified knowledge on self-care and the motivation that involves supervision and “peer-to-peer” community work.

This contrasts the results reported by Wang et al. (2012); in their study, conducted in older adults with MS aged 70 to 89 years, they compared the training effect of the combination of aerobic, strength, balance, and flexibility exercises (*n* = 181) versus an education program for successful aging for 12 months and observed a decrease of less than 15% in the prevalence of MS at 6 months after the intervention, without statistically significant differences between the groups [37]. The difference between the effect of this study and that of our findings may be due to the characteristics of the gerontology program, in which empowerment and social support networks among peers are key elements.

In this regard, one of the best-known self-care programs aimed at older adults is the “Living with Vitality”^®^ program developed in 1996 at the Autonomous University of Madrid by the Fernández-Ballesteros research group, demonstrating the positive effect of knowledge of the self-care of the elderly through a formal, “face-to-face” course, as well as in multimedia and distance versions, by the adoption of healthy lifestyles and a positive perception of aging and old age, which has been applied in the Mexican population [38,39,40,41]. Likewise, the model developed by our research group has demonstrated its effect on the improvement in cognitive function, physical functionality, and oxidative stress [21,22,23], for which we consider that it could be implemented to promote healthy aging for the general population of older adults or groups with specific problems, as has been shown for the control of MS.

In the individual analysis of the changes in MS parameters, we observed a statistically significant decrease in BMI and blood pressure in the group that participated in the training program for self-care. In this sense, our findings agree with those reported in other community intervention studies, where it has been shown that practicing healthy lifestyles for more than 3 months has a positive effect on BMI and blood pressure [42,43].

Moreover, a statistically significant increase in the blood concentration of HDL was observed in EG, compared with CG. This effect has also been observed in community programs and training programs for physical exercise at home and modification of eating habits [42,44].

Regarding triglyceride concentrations, a statistically significant decrease was found in EG, compared with CG, which is consistent with that reported in studies on the impact of physical exercise and diet on MS [44,45]. This effect is related to the changes found in overweight and obesity, in addition to the inverse relationship between triglyceride levels and HDL.

The effect of the intervention program on the specific components of MS, the highest percentage of change was found in the parameters of DBP (−34%), SBP (−33%), HDL (−27%), and triglycerides (−11%). In this regard, the study carried out by Ortiz-Rodríguez et al. (2017) on the prevalence of MS in the Mexican population of older adults report that the criteria with the highest frequency detected were arterial hypertension (92.4%), hypertriglyceridemia (77.8%), low HDL levels (77.1%), hyperglycemia (71.1%), and central obesity (65.4%) [9].

As can be seen, there is a coincidence in three of the parameters, where the greatest effect was achieved with the community program implemented in this research, which would support the proposal that this program can be considered as an effective and low-cost option among community programs for the prevention and control of MS in older adults. For this reason, the effect of CGP could have an impact on the main components of MS prevalent in our population.

Among the perspectives for future studies focused on the prevention of MS during aging, we must consider the influence of eating habits, such as the consumption of fruits and vegetables and high-calorie intake during dinner [45,46], the gut microbiota profile [47], the characteristics of social support networks [48], socioeconomic inequalities, age and gender [49], as well as the relationship and prognosis of the components of the metabolic syndrome with successful aging [50].

Among the main limitations of this study, it is important to highlight that the size and characteristics of the sample were not representative, so the results cannot be generalized; in addition, the short intervention period did not allow observing the effect on some relevant parameters of MS such as central obesity and hyperglycemia. The influence of other factors such as diet and sleep as contributing variables was also not accurately assessed. For this reason, it is beneficial to carry out more studies with representative samples in different sociocultural contexts and establish strategies for monitoring the type of diet, physical activity, time, and quality of sleep in both groups.

## 5. Conclusions

This study supports the proposal of the WHO (2015) regarding the development and implementation of active aging models with community gerontology programs [36], to strengthen empowerment and the formation of a network of social support networks between peers as social capital for healthy aging [51,52]. Our findings demonstrated 83% MS control in older adults who participated in the CGP. The main components of MS that showed improvement were high diastolic and systolic blood pressure and low blood concentration of HDL.

## Figures and Tables

**Table 1:** Topics of the community gerontology program for healthy aging.

	Topic	Activity	Sessions per Week	Hours per Session	Total Sessions	Total Hours
1	Active and healthy aging	Theoretical/debate	1	2	3	6
2	Healthy eating	Theoretical/practical	1	2	3	6
3	Safe physical exercise	Theoretical/practical	1	2	3	6
4	Personal and environmental hygiene	Theoretical/practical	1	2	2	4
5	Sleep hygiene	Theoretical/debate	1	2	2	4
6	Self-esteem vs. ageism	Theoretical/debate	1	2	2	4
7	Leisure and recreation	Theoretical/practical	1	2	2	4
8	Self-care for healthy aging	Theoretical/practical	1	2	3	6
					20	40

**Table 2:** Sociodemographic characteristics by study group.

	**Control Group** ***n* = 30**	**Experimental Group** ***n* = 36**
Age (mean ± SD)	71 ± 9	69 ± 8
Sex (%)		
Men	10 (33)	8 (22)
Women	20 (67)	28 (78)
Civil status (%)		
With couple	9 (30)	12 (33)
Without couple	21 (70)	24 (67)
Scholarship (years)	7 ± 3	6 ± 3
Socioeconomic level (%)		
Low	19 (63)	25 (70)
Medium	7 (23)	8 (22)
High	4 (14)	3 (8)

Chi-squared and Student’s *t*-tests; *p* > 0.05.

**Table 3:** Mean numbers of MS diagnostic criteria pre- and post-intervention.

Measurement	Control Group *n* = 30 (%)	Experimental Group *n* = 36 (%)	*p* Value
Pre-intervention	3.1 ± 0.4	3.2 ± 0.5	
Post-intervention	3.5 ± 0.9	2.0 ± 1.2	<0.001

Data presented are means and standard deviation. Repeated measures ANOVA, 95% significance.

**Table 4:** Pre- and post-intervention clinical parameters by group.

	Control Group *n* = 30	Experimental Group *n* = 36	*p* Value
Weight (kg)			
Pre-intervention	68 ± 12	71 ± 20	
Post-intervention	71 ± 10	66 ± 11	0.792
Height (cm)			
Pre-intervention	158 ± 11	148 ± 8	
Post-intervention	157 ± 10	148 ± 7	0.997
BMI (weight/height^2^)			
Pre-intervention	28 ± 5	32 ± 6	
Post-intervention	29 ± 3	30 ± 5	0.009
Waist (cm)			
Pre-intervention	99 ± 10	104 ± 12	
Post-intervention	98 ± 9	102 ± 15	0.142
Hip (cm)			
Pre-intervention	103 ± 10	108 ± 14	
Post-intervention	104 ± 10	105 ± 13	0.190
SBP (mm Hg)			
Pre-intervention	128 ± 16	129 ± 14	
Post-intervention	132 ± 10	126 ± 20	0.047
DBP (mm Hg)			
Pre-intervention	82 ± 8	85 ± 11	
Post-intervention	85 ± 9	81 ± 13	0.048

Abbreviations: BMI, Body mass index; SBP, systolic blood pressure; DBP, diastolic blood pressure. Data presented are means and standard deviation. Repeated measures ANOVA, 95% significance.

**Table 5:** Pre and post-intervention biochemical parameters by group.

	Control Group *n* = 30	Experimental Group *n* = 36	*p* Value
Glucose (mg/dL)			
Pre-intervention	118 ± 31	111 ± 20	
Post-intervention	124 ± 45	112 ± 32	0.164
Urea (mg/dL)			
Pre-intervention	35 ± 7	35 ± 9	
Post-intervention	33 ± 8	37 ± 10	0.246
Urate (mg/dL)			
Pre-intervention	5 ± 1	5 ± 2	
Post-intervention	6 ± 2	4 ± 1	0.080
Cholesterol (mg/dL)			
Pre-intervention	206 ± 57	207 ± 44	
Post-intervention	211 ± 38	185 ± 19	0.140
HDL (mg/dL)			
Pre-intervention	52 ± 12	56 ± 16	
Post-intervention	46 ± 9	63 ± 17	0.001
Triglycerides (mg/dL)			
Pre-intervention	192 ± 78	177 ± 61	
Post-intervention	200 ± 90	139 ± 43	0.004
Albumin (mg/dL)			
Pre-intervention	4.5 ± 0.23	4.5 ± 0.52	
Post-intervention	4.3 ± 0.51	4.3 ± 0.52	0.835
Creatinine (mg/dL)			
Pre-intervention	0.91 ± 0.48	0.78 ± 0.20	
Post-intervention	0.78 ± 0.25	0.76 ± 0.32	0.207
CRP (mg/dL)			
Pre-intervention	0.28 ± 0.39	0.47 ± 0.38	
Post-intervention	0.30 ± 0.47	0.41 ± 0.31	0.057

Abbreviations: HDL, high-density lipoprotein; CRP, C-reactive protein. Data presented are means and standard deviation. Repeated-measures ANOVA, 95% significance.

**Table 6:** Frequency of metabolic syndrome criteria pre- and post-intervention by group.

MS Criteria	Control Group *n* = 30 (%)	Difference	Experimental Group *n* = 36 (%)	Difference	*p* Value
Abdominal circumference *		(5)		(−9)	
Pre-intervention	23 (78)		33 (92)		
Post-intervention	25 (83)		29 (81)		0.125
Glucose ≥ 110 mg/dL		(0)		(−9)	
Pre-intervention	17 (57)		19 (53)		
Post-intervention	17 (57)		15 (42)		0.267
SBP ≥ 130 mm Hg		(13)		(−33)	
Pre-intervention	14 (47)		22 (61)		
Post-intervention	18 (60)		10 (28)		0.004
DBP ≥ 85 mm Hg		(7)		(−34)	
Pre-intervention	18 (60)		20 (56)		
Post-intervention	20 (67)		8 (22)		0.006
Triglycerides ≥ 150 mg/dL		(−7)		(−11)	
Pre-intervention	26 (87)		27 (75)		
Post-intervention	24 (80)		13 (64)		0.002
HDL *		(27)		(−27)	
Pre-intervention	13 (43)		16 (44)		
Post-intervention	21 (70)		6 (17)		0.031

Abbreviations: MS, metabolic syndrome; SBP, systolic blood pressure; DBP, diastolic blood pressure. Abdominal circumference: * Men ≥ 102 cm, Women ≥ 88 cm, HDL, * high-density lipoprotein: men ≤ 40 mg/dL, women ≤ 50 mg/dL. Data presented are frequencies and percentages. McNemar test for related samples, 95% significance.

## Data Availability

The data presented in this study are available on request from the corresponding author.
